# Cigarette Smoking and Dyspnea Perception

**DOI:** 10.1186/1617-9625-2-3

**Published:** 2004-03-15

**Authors:** Elisabetta Rosi, Giorgio Scano

**Affiliations:** 1 Section of Immunoallergology and Respiratory Diseases, Department of Internal Medicine, University of Florence, Firenze, Italy

## Abstract

Cigarette smoking has been implicated as an important risk factor for the development of respiratory symptoms in adults. The relationship of dyspnea with cigarette smoking has been examined in smokers and ex-smokers and the beneficial effects of smoking cessation have been demonstrated. Recent studies reported that in subjects who smoke cigarettes the risk of developing respiratory symptoms is higher in a dose-dependent way. Environmental tobacco smoke heavily influences the incidence of respiratory symptoms in both adults and in children. Up to the present time, the mechanisms whereby cigarette smoking causes dyspnea perception remain to be defined. Abnormalities in sensory nerves might diminish the perception of bronchoconstriction in smokers. In this regard, it has been postulated that prolonged exposure to cigarette smoke may lead to chronic depletion of sensory nerve neurotransmitters. Eosinophil airway inflammation has been proposed as a determinant of breathlessness via mechanisms affecting either the mechanical pathways that control breathlessness or the afferent nerves involved in perception of dyspnea. An increased number of eosinophils in some smokers implies the possibility that smoking may trigger immunological or other reactions associated with eosinophilia. In conclusion, cigarette smoking is by far one of the greatest risk factors for most respiratory symptoms, including dyspnea. Smoking is associated with the development of symptoms in a dose-dependent way and eosinophilia and airway hyperresponsiveness (AHR) increase the risk of developing dyspnea.

## Introduction

The mechanisms responsible for inter-individual differences in dyspnea perception are difficult to clarify due to the wide variation in the perception of breathlessness present in normal subjects [1] in whom the experience of breathlessness may modify subsequent estimates of the symptom [2]. In a disease state, it may not be possible to identify these inherent inter-individual differences and this may confound attempts to identify the effects due to disease. Mechanical factors including respiratory muscle activity [3] associated with pulmonary hyperinflation [4-6], temporal adaptation [7-9] and bronchial hyperresponsiveness [7], psychological factors [10] or emotional and cognitive factors [11] have been proposed to influence the perception of dyspnea. Moreover, some recent reports [12-16] make a strong case for airway inflammation contributing to cover a part of the unexplained variability of the symptom.

## Cigarette Smoking and Respiratory Symptoms

Cigarette smoking has been implicated as an important risk factor for the development of respiratory symptoms in adults [17]. On this topic significant results have been obtained from the group of Tucson. Krzyzanowski and Lebowitz [17] showed that subjects who continued to smoke during the 11 to 13 years of follow-up ran a two or three times higher risk of developing respiratory symptoms such as dyspnea and attacks of breathlessness compared with lifetime non-smokers. Krzyzanowski and Lebowitz [17] examined data from two longitudinal studies conducted in Cra-cow, Poland, and Tucson, Arizona, to assess the similarities in the relationship of symptoms to age and smoking habit in the two cities. The relationship of symptoms to smoking was similar in both cities, after adjustment for age and gender, with at least doubled incidence rates of most symptoms in continuous smokers compared to lifetime non-smokers. Furthermore, the same authors [18] investigated the relationship between persistence and incidence rates of respiratory symptoms and smoking cessation. Among 1,722 subjects smoking at the beginning of the study, 468 had given up smoking by the end of the 13-year follow-up. The persistence and incidence rate of attacks of breathlessness as well as chronic cough, chronic phlegm and wheeze were reduced by 50% in ex-smokers compared to those subjects who continued to smoke. The beneficial effects of smoking cessation were lower in those subjects who had smoked a higher number of cigarettes per day in the past and had started smoking younger. The symptoms were less likely if smoking ceased before the onset of any respiratory disease. These results were similar in the Cracow and Tucson populations, suggesting the universal nature of the observations [18]. Smokers who are able to break the habit generally experience a reduction in respiratory symptoms and improvement in pulmonary function; however, gender and particularly age are important influencing factors [19,20]. Sherril and colleagues [19], analyzing respiratory function and symptom data from a follow-up period of up to 20 years in Tucson, compared the respiratory status between restarters, consistent smokers and ex-smokers. The symptoms included any wheeze, cough, dyspnea or phlegm. The authors demonstrated that there were no statistically significant differences between the initial symptoms reported by restarters and consistent smokers. However, consistent smokers did report significantly more dyspnea during the last survey.

Data were similar for male and female subjects. The results of the symptom-analysis of both the initial and last surveys would suggest that although restarters have the steepest rates of decline in FEV_1_, they have fewer symptoms than consistent smokers who reported more dyspnea at the last survey [19]. This might arise from a reporting bias by subjects who relapsed, being less likely to admit symptoms resulting from their inability to quit the habit even if they reported more phlegm. The fact that male ex-smokers had more symptoms than restarters implicates that this subgroup contains subjects who quit the habit after developing symptoms that, for the most part, were irreversible [21]. More recently, Jansen and colleagues [22] (see Table 1) found that cigarette smokers had an increased risk of developing respiratory symptoms compared to people who had never smoked. They demonstrated, through a 25-year follow-up study of the general adult population from Vlagtwedde and Vlaardingen (The Nederlands), that in subjects who smoke cigarettes the risk of developing respiratory symptoms is higher in a dose-dependent way. Dyspnea perception was assessed using the Dutch version of the British Medical Research Council (MRC) standardized questionnaire. Subjects were considered symptomatically affected if their reported dyspnea was over grade 3 of the MRC questionnaire. Development of wheeze and dyspnea appeared to occur especially in subjects with both airway hyperresponsiveness (AHR) and peripheral blood eosinophilia [22]. On the contrary Xu and colleagues [23] have shown that the risk of smoking for the development of symptoms was the same in AHR+ as in AHR- subjects. Lindström and colleagues [24] (see Table 1) performed a comparative study between Northern Sweden and Northern Finland by analyzing the influence of smoking on the prevalence of respiratory symptoms including exertional dyspnea. Their results indicate that the prevalence of symptoms is strongly related to the number of cigarettes smoked per day. Furthermore, they observed that bronchitic symptoms were more common in Finland, even after the correction of demographic variables including smoking habits. According to the authors, this difference may be due to air pollution and particularly to environmental tobacco smoke (ETS).

**Table 1:** Prevalence of development of respiratory symptoms by smoking habits

**From Jansen et al. (8)**		**OR (respiratory symptoms)**	
	Ex smoker	1.21	
	1–14 cig/day	1.89*	
	15–24 cig/day	2.98*	
	>24 cig/day	3.57*	
**From Lindström et al. (10)**		**% (Dyspnea grade 3)-Norrbotten**	**% (Dyspnea grade 3) Lapland**
	Nonsmokers	4.5 (m) 7.7(f)*	8.1(m) 17.2(f)*
	Ex-smokers	46.7 (m) 12.8(f)*	18.5 (m) 20.4(f)*
	smokers	10.3 (m) 11.7(f)*	12.7 (m) 18.2(f)*

*p < 0.001; f: female, m: male

## Dyspnea and Environmental Tobacco Smoke

It has been demonstrated that ETS heavily influences the incidence of respiratory symptoms both in adults [25,26] and in children [27]. Jaakkola and colleagues [25] examined the relationship between exposure to ETS and development of respiratory symptoms including dyspnea, in a population of 117 subjects who had never smoked and who were aged between 15 and 40 at the time of initial examination, and who were reexamined eight years later. A significant dose-related increase in the risk of developing dyspnea was observed in connection with ETS exposure. In a recent paper Gilliland and colleagues [28] demonstrated that maternal smoking during pregnancy and current ETS exposure were associated with an increased prevalence of attacks of wheezing causing shortness of breath in children residing in 12 communities in Southern California.

Jansen and colleagues [22] (see Table 1) recently demonstrated that among current smokers the number of cigarettes smoked per day was consistently the strongest risk factor relative to the development of chronic cough, chronic phlegm, wheeze, and dyspnea respectively. Schenker and colleagues observed in adult women that cigarette particulate content (tar) was significantly related to cough and phlegm production, while the association between smoking and symptoms of wheeze and dyspnea was supposed to be more strongly related to the vapor phase of cigarette smoke [29]. In a more recent study, after adjustment for intensity and length of smoking history and for depth of inhalation, the risk of chronic phlegm, cough, and dyspnea were not related to the tar and nicotine yields [30].

**Figure 1:**
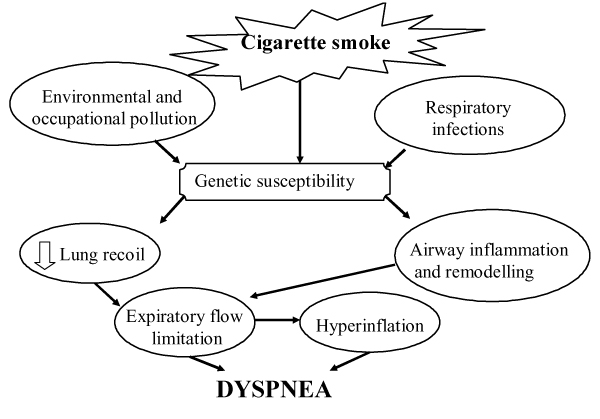
**Pathogenetic mechanisms of dyspnea in smokers with COPD**. Exposure to cigarette smoke is the major factor in the pathogenesis of COPD. Risks conferred by smoking interact with genetic susceptibility and respiratory infections to produce expiratory flow limitation (EFL) by loss of elastic recoil and airway inflammation. EFL, by producing hyperinflation and hyperinflation per se generate dyspnea sensation.

Up to the present time, the mechanisms whereby cigarette smoking causes dyspnea perception remain to be defined. Figure 1 is an attempt to elucidate some potential mechanisms (for explanation see below). Abnormalities in sensory nerves might diminish the perception of bronchoconstriction in smokers. In this regard, it has been postulated that prolonged exposure to cigarette smoke may lead to chronic depletion of sensory nerve neurotransmitters [31].

## Cigarette Smoking and Copd

Active smoking is certainly the most important causative factor of COPD (Figure 1), even though only a proportion of smokers will develop the disease. There is consisting evidence of a dose-response relationship between the amount of smoking and the decline in FEV_1 _[32]. Regarding the reversion of smoking effect on bronchial obstruction, a reduction in the decline of FEV_1 _has been demonstrated, without returning to the basal level. [33]

**Figure 2:**
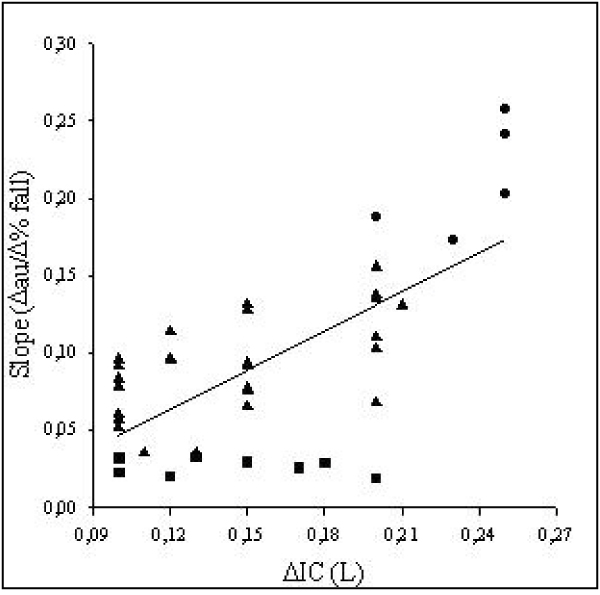
**Relationship between change (Δ) in inspiratory capacity (IC) and slope of Borg score on FEV1% fall during methacholine-induced bronchoconstriction**. Symbols indicate: square – hypoperceivers; triangles – normoperceivers; circles – hyperperceivers. (Reproduced with permission from reference [36].) For explanation see text.

**Figure 3:**
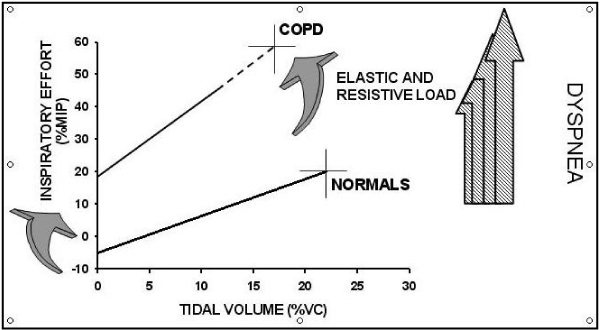
**This shows the increase in inspiratory effort for any given tidal volume (VT)**. The curved arrows indicate the increase in both slope (b) and intercept (a) of the VT to inspiratory effort relationship in patients as compared to healthy subjects. The straight arrows show the progressive increase in dyspnea with increasing b and a (for explanation see text). Abbreviations: MIP = maximal inspiratory pressure; VC = vital capacity; COPD = chronic obstructive pulmonary disease.

Smoking habit has a prominent role in the induction of chronic bronchial obstruction leading to COPD and the major symptom of the disease [34]. (Figure 1). Despite similar level of bronchoconstriction the degree of dyspnea sensation may be widely variable in COPD [35,36] as well as in asthmatic patients [16,37,38]. Getting insight into these mechanisms, Ottanelli and colleagues [36] have shown that smokers with chronic airflow limitation could be either hypoperceivers, moderate perceivers or hyperperceivers to an acute change in airway caliber with methacholine. In 39 patients dyspnea was assessed during a methacholine-induced FEV_1 _% fall using a Borg scale. Acute hyperinflation accounted in part for the variability in the perception of dyspnea after accounting for change in FEV_1 _during bronchoconstriction. (Figure 2). In that, Ottanelli and colleagues [36], in line with previous data [35,37], have identified some potential factors for increasing the prediction of the variation in breathlessness after accounting for FEV_1 _decrease. The increase in end expiratory lung volume on the one hand increases the central output to the respiratory muscles and on the other decreases their maximal capacity of generating inspiratory pressure. That increases the sense of inspiratory effort, that is, breathlessness. Furthermore, because of the bronchoconstriction and expiratory flow limitation, the increased respiratory drive does not result in a proportional increase in tidal volume or inspiratory flow. This is another potential mechanism of dyspnea [34]. As shown in Figure 3, for a given tidal volume a greater inspiratory effort is needed in smokers with airflow limitation as compared to healthy subjects, such that the greater the slope (b) of VT to effort relationship, the greater the perception of dyspnea. Figure 3 also shows the increase in the intercept (a) of this relationship, which defines the increase in end expiratory lung volume (hyperinflation). Thus, both a and b result in an increased dyspnea sensation.

## Cigarette Smoking and Airway Inflammation

**Figure 4:**
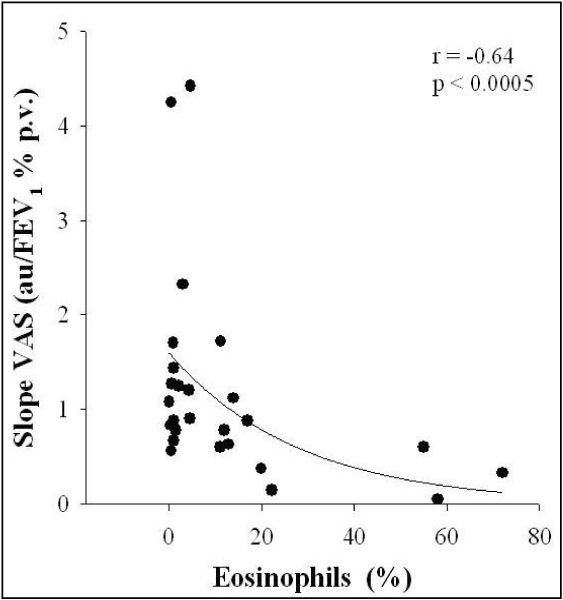
**Relationship of the level of sputum eosinophils with perception of dyspnea during bronchoconstriction assessed as slope of changes in VAS on FEV1 decrease during methacholine challenge (Reproduced with permission from reference **[16].

Neutrophilic airway inflammation and release of neutrophil chemoattractant cytokines, including IL-8, have been observed to be associated with smoke exposure, with sputum IL-8 concentration being related to percent-predicted forced expiratory volume in 1 second (FEV_1_) in smoking asthmatics [39]. However, the influence of neutrophilic airway inflammation on respiratory symptoms has not yet been determined [39,40]. On the other hand, eosinophil airway inflammation has been proposed as a determinant of breathlessness [41-43] via mechanisms affecting either the mechanical pathways that control breathlessness [44,45] or the afferent nerves involved in perception of dyspnea [16,41]. Ottanelli and colleagues [16] have shown that eosinophilic inflammation of the airway wall may affect the perception of dyspnea positively and that the association of eosinophils with inhaled corticosteroids may result in the perception of dyspnea in asthma getting worse (Figure 4). Two cases of acute eosinophilic pneumonia associated with cigarette smoking, after excluding any other identifiable cause, have recently been reported [46]. Mensinga and colleagues [47] examined the independent and combined effect of skin test reactivity and eosinophilia on the prevalence of a variety of respiratory symptoms, after adjusting for age, sex, smoking habit, and area of residence. Subjects with eosinophilia were more likely to be symptomatic than subjects without eosinophilia with the exception of male subjects who never smoked [47]. The total number of leukocytes may be increased by cigarette smoking [48]. One of the components of the leukocyte count is formed by the overall number of eosinophils. An increased number of eosinophils in some smokers implies the possibility that smoking may trigger immunological or other reactions associated with eosinophilia [49]. Nevertheless, Jansen and colleagues [22] demonstrated that peripheral blood eosinophilia increased the risk of developing dyspnea independent of smoking in the general adult population.

The presence of respiratory symptoms has been examined as a marker of susceptibility to the detrimental effect of smoking on respiratory function. In an analysis from the Tucson study of data on the growth of pulmonary function between 5.5 and 25 years of age, symptoms and smoking had a negative impact on growth of lung function, using FVC, FEV_1_, Vmax50, and size-compensated flows (Vmax50/FVC). Smoking cessation was shown to have a positive impact on growth of pulmonary function. Young smokers without respiratory symptoms experienced the same longitudinal changes in FEV_1 _as non-smokers [50].

Interestingly, the habit of smoking may influence dyspnea perception also in fibrotic diseases. De Cremoux and colleagues [51] examined two populations of patients with idiopathic pulmonary fibrosis: non-smokers versus smokers. Despite similar pulmonary function test results, non-smokers had a shorter duration of symptoms at the time of presentation than smokers. Prednisolone therapy was more efficient in non-smokers than in smokers.

In summary, cigarette smoking is by far one of the greatest risk factors for most respiratory symptoms, including dyspnea. Smoking is associated with the development of symptoms in a dose-dependent way and eosinophilia and AHR increase the risk of developing dyspnea. In short, further studies are necessary in order to establish the exact interaction between cigarette smoking and airway inflammation with dyspnea perception.

## Competing interests

The authors declare that they have no competing interests.
